# Clear aligner’s adverse effects: A systematic review protocol

**DOI:** 10.1371/journal.pone.0302049

**Published:** 2024-05-02

**Authors:** Cintia Ronchi Lemos, Marianella Aguilar Ventura Fadel, Helena Polmann, Júlia Meller Dias de Oliveira, Patrícia Pauletto, Cristine Miron Stefani, Carlos Flores-Mir, Graziela De Luca Canto

**Affiliations:** 1 Brazilian Centre for Evidence-Based Research (COBE), Department of Dentistry, Federal University of Santa Catarina (UFSC), Florianópolis, Brazil; 2 School of Dentistry, Universidad De Las Américas (UDLA), Quito, Ecuador; 3 Brazilian Centre for Evidence-Based Research (COBE), University of Brasilia (UNB), Brasília, Brazil; 4 Department of Dentistry, University of Alberta, Edmonton, Canada; Justus Liebig University Giessen, GERMANY

## Abstract

With the growing popularity of clear aligners, more patients have chosen to use them instead of traditional orthodontic braces to correct their malocclusions. Clear aligners offer distinct advantages over conventional fixed braces, such as limited aesthetic impact, the convenience of easily removing them for meals, improved accessibility for brushing and flossing, and a treatment approach that avoids the use of metal, minimizing potential irritation to the cheeks and gums. Manufacturers point out a disadvantage that can be administered in this type of treatment. Still, to our knowledge, a comprehensive review of the published literature assessing the adverse/negative effects of clear aligners has not yet been conducted. A systematic review, with or without meta-analysis, will be performed. The inclusion criteria will be studies involving individuals using clear aligners and reporting potential adverse/negative effects during or after treatment. No restrictions about time or language will be applied. The studies screening will be conducted in two stages. Two independent reviewers will initially evaluate the title and abstract under the eligibility criteria. Subsequently, the same two reviewers will examine the articles’ full text in-depth. The results will be synthesized in the form of a narrative description and incorporate a meta-analysis if justified. Furthermore, we will present details regarding the sample characteristics, intervention, study objectives, methodologies employed, and primary findings. This study aims to investigate the potential adverse effects and their frequency among orthodontic patients wearing clear aligners. Moreover, the outcomes of this review have the potential to illuminate specific inherent limitations of aligner therapy as a comprehensive orthodontic approach.

## Background

The constant quest for a perfect smile and correcting orthodontic issues have led many patients and healthcare professionals to consider alternatives to traditional fixed orthodontic appliances. Clear aligners are effective for milder malocclusions but face challenges in achieving outcomes comparable to fixed appliances in more complex cases. Time efficiency varies based on the orthodontist’s experience and the number of cases treated, with aligners showing superior results in aspects like dental alignment and treatment duration for milder malocclusions [1–4]. In this context, clear aligners have gained prominence as a more aesthetic and perceived comfortable option. Clear aligners are made of transparent material, almost imperceptible during use, making them a much more attractive option than metallic fixed appliance systems. They offer an inherently more appealing alternative for individuals seeking to address dental malocclusion without compromising aesthetics [1, 2, 5]. However, as the popularity of these devices grows, it becomes crucial to examine their benefits and the potential adverse effects associated with their use.

A previous study [5] alerted to the possibility of serious adverse events, some of which can be life-threatening, such as difficulty breathing, swollen throat, anaphylactic reaction, swollen lips, laryngeal spasms, blisters, etc., during clear aligner treatment. This leads to how much awareness healthcare professionals have of these events and whether they are prepared to deal with them. Open and transparent communication between professionals and patients is essential, allowing the latter to make informed decisions about their orthodontic therapy [5].

Additionally, periodontal health during aligner treatment is a relevant topic, highlighted by various research studies [6–9]. While aligners may provide better biofilm control than fixed appliances, the differences are generally considered clinically insignificant. However, the incidence of gingival recession and white spot lesions is a concern, and the choice between aligners and fixed appliances should be based on the individual patient’s needs and risks [8–12]. Furthermore, clear aligners can affect speech articulation, with significant implications for patients whose professions depend on clear and accurate speech [13]. In a systematic review examining patients’ perceptions of clear aligner treatments, the authors recognized the necessity for additional studies investigating subjective outcomes compared to traditional devices. Particularly, there is a call for studies addressing challenges in speaking, chewing, sleeping, and discomfort associated with clear aligners [14].

The published systematic reviews focus on specific adverse events, such as apical root resorption or periodontal injuries. This study aims to aggregate all types of adverse events reported in the literature [8, 15]. Given the significance of this information, combined with over 17 million cases treated, according to data from the world’s largest clear aligner manufacturer [16], the objective of this systematic review is to answer the following specific question: What are the potential real and perceived adverse effects associated with orthodontic treatment using clear aligners?

## Methods

### Protocol and registration

This protocol was developed according to the Preferred Reporting Items for Systematic Reviews and Meta-Analyses Protocols (PRISMA-P) (S1 Appendix). The protocol has been registered in the International Prospective Register of Systematic Reviews (PROSPERO) under number: CRD42023458491 [17–19].

### Research question

This systematic review seeks to answer the question: “What are the potential adverse effects associated with orthodontic treatment using clear aligners?”. The research question is based on the PIOS acronym (Table 1) [17, 18, 20–22].

**Table 1:** Research question based on PIO acronym.

**P**	PARTICIPANTS	Orthodontic patients regardless of age and sex.
**I**	INTERVENTION	Clear aligner treatment.
**O**	OUTCOMES	Any type of adverse/negative effects—any harmful and undesirable effect resulting in medication or intervention need.
**S**	STUDY DESIGN	Clinical trials randomized or not, before-and-after studies, cohort, or descriptive studies.

### Eligibility criteria

#### Inclusion criteria

It will include articles that fit the following eligibility criteria:

- Studies with orthodontic patients regardless of their age or sex;
- Studies that assessed any kind of clear aligners and which reported possible adverse effects during and/or after treatment;
- Randomized clinical trials, non-randomized clinical trials, quasi-randomized clinical trials (before and after), cohort studies, and descriptive studies.

Note: In the case of randomized and non-randomized clinical studies, information will be collected from the group of patients who used the clear aligner systems (single arm).

#### Exclusion criteria

The following studies will be excluded:

- Studies in which patients were treated using a hybrid approach (clear aligners and another orthodontic approach during the same treatment);
- Reviews, letters, books, conference abstracts, case reports, opinion articles, technique articles, posters, and clinical practice guidelines;
- Full text is unavailable, even after contacting the corresponding authors (three attempts in 3 weeks).

#### Information sources and search strategy

A preliminary search strategy was developed and refined with the librarian’s assistance. Subsequently, to identify pertinent research, a comprehensive search strategy was developed and implemented across appropriate databases to retrieve potentially relevant studies (S1 and S2 Appendices). With the support of a specialized librarian in health sciences, a systematic search will be executed in the following databases: Cochrane (Central), EMBASE, LILACS (in Spanish: *Literatura Latinoamericana y del Caribe en Ciencias de la Salud*), LIVIVO, PubMed/MEDLINE, SCOPUS, and Web of Science. The grey literature will be consulted through Google Scholar and ProQuest Dissertations & Theses Global. The citation list of the included studies and experts in the subject will still be consulted. The search will be done in January 2024 by the first author (C.R.L.) [17, 22].

#### Study records

*Data management*. The files from each database will be collected and imported into a reference software manager (EndNote-Web^™^, Clarivate^™^, Jersey, USA). Duplicates will be removed in the same software. Then, the selected studies will be imported into a reading platform (Rayyan^®^, Qatar Computing Research Institute, Data Analytics, Doha, Qatar) [17–19, 23, 24].

#### Methods for study selection

The study will be selected in two phases with two calibrated and independent reviewers (C.R.L. and M.A.V.F.). The inclusion and exclusion criteria will be applied to guide this process. The two reviewers (C.R.L. and M.A.V.F.) will read the titles and abstract of the selected articles in phase 1. In phase two, the same reviewers (C.R.L. and M.A.V.F.) will read the full texts of the previously included studies. The same criteria as the first selection stage will be used. In disagreement, a third reviewer (H.P.) will decide [17, 19, 22, 23].

#### Data collection process

The data of the included articles will be extracted by two independent reviewers (C.R.L. and M.A.V.F.) in a Microsoft Excel^®^ (Microsoft Corporation, Redmond, WA, USA) table form. Any divergences of the reviewer will be resolved by a third discussion or with a fourth reviewer (H.P.). If necessary, data transformation will be performed, such as the conversion of odds ratio (OR) to relative risk (RR) or graphical data extraction using the WebPlotDigitizer software (https://automeris.io/WebPlotDigitizer/). The extracted data will be presented in the article in a table outlining the characteristics of the included studies. In cases where two or more strict inclusions originate from the same study, the articles will be collectively analyzed. Data will be extracted from each article based on the follow-up period. The collected data will be organized into a table containing all the characteristics of the included studies [17, 19, 22, 23].

#### Data items

From the studies found, the following data will be collected: Authors, country, year, sample size, groups (with clear aligners/without clear aligners or other type of orthodontic appliances), type of malocclusion, clear aligner brand, orthodontic treatment duration, adverse effects type and frequency, detection method criteria for adverse effects, results, main conclusion, and study type [17, 19, 22, 23].

#### Outcomes and prioritization

Any adverse effect that is a harmful and undesirable effect resulting in medication or intervention, such as some initial discomfort while wearing the aligners, speaking impairment—especially in the first few days, excessive salivation, white spot lesions, root resorption, and bruxism. The outcome can be measured by any method, such as questionnaires, complementary exams, or clinical assessment. They will be measured by using frequency measures [17, 19, 22, 23].

#### Risk of bias in individual studies

Two independent reviewers (C.R.L. and M.A.V.F.) will assess the risk of bias in the included studies. The tools used will be chosen according to the type of study included. RoB 2.0 (www.riskofbias.info) for randomized clinical trials, ROBINS-I for non-randomized intervention studies, and ROBINS-E for observational studies. Differences in the evaluation will be resolved by a third reviewer (H.P.).

Before applying the tool, the authors will discuss it and establish the parameters for its evaluation. After that, a calibration between the evaluators will also be carried out. The results will be presented as recommended by each tool. Figures will be created on the *robvis* website (https://www.riskofbias.info/welcome/robvis-visualization-tool) [17, 19, 22, 23].

#### Data synthesis

The research team plans to use the R software to conduct a proportion meta-analysis and subgroup analysis (brand, time of use, material). Clinical and methodological considerations will be applied before deciding the pertinence of meta-analyses. Effect sizes with a 95% confidence interval will be calculated using random-effects models for the overall meta-analysis and subgroup analyses. Heterogeneity will be assessed using the I^2^ statistics and the Cochran’s Q test [15]. A P value lower than 5% provides evidence of heterogeneity of intervention effects.

#### Meta-bias(es)

An exhaustive literature search will be conducted to prevent potential publication bias. In addition, to uphold the research’s integrity and impartiality, affiliations of the sponsors associated with the included studies will be assessed, and any potential conflicts of interest among the authors will be scrutinized. If more than ten studies are included, publication bias, if present, will be assessed using the funnel plot method.

#### Confidence in cumulative evidence

Considering the descriptive nature of the data and the type of planned meta-analysis (proportions), the certainty of evidence evaluation will not be performed in this review. Statistical heterogeneity will be assessed among the results of different trials using the Chi-square (Chi^2^) test, with significance defined as P<0.1. The I^2^ statistic will be employed to evaluate heterogeneity within studies in each analysis. Heterogeneity will be categorized as ’not important’ (0% to 40%); ’moderate’ (30% to 60%); ’substantial’ (50% to 90%); and ’considerable’ (75% to 100%). Additionally, heterogeneity will be assessed by visually inspecting the overlap of confidence intervals (CIs) and may be explored in subgroup analyses if applicable. In the case of substantial heterogeneity, the impact of high-risk-of-bias studies and studies with small sample sizes on the overall effect estimate will be explored by removing studies with these characteristics from each meta-analysis [17, 19, 22].

## Discussion

This systematic review aims to meticulously examine the existing evidence concerning adverse effects linked to clear aligner use. The study is distinguished by its robust methodology, encompassing a thorough search across all available literature in six databases, along with grey literature, reference lists, and collaboration with field experts, enhancing the breadth and reliability of the results. Methodological transparency facilitates reproducibility, allowing other researchers to independently validate the findings. To ensure study quality and prevent selection bias, the review employs two blinded reviewers supported by a third reviewer in the decision-making process. The comprehensive analysis offers valuable insights to consumers contemplating orthodontic treatment, potentially revealing undisclosed adverse effects that could influence the attractiveness of clear aligners for individuals and also improve the provided information during the informed consent process. Despite certain limitations, such as potential bias risks and the risk of bias of the studies to be included, this review can serve as a valuable resource and contribute to a comprehensive understanding of adverse events associated with clear aligners. Promoting a balanced perspective in the ongoing debate about orthodontic treatment choices, this will benefit both professionals and patients, guiding them in making informed decisions regarding this form of orthodontic treatment.

## Supporting information

S1 Appendix PRISMA-P (Preferred Reporting Items for Systematic review and Meta-Analysis Protocols) 2015 checklist: Recommended items to address in a systematic review protocol*. (DOCX)

S2 Appendix (DOCX)

## Abbreviations

LILACS: Literatura Latinoamericana y del Caribe en Ciencias de la Salud
PRISMA-P: Preferred Reporting Items for Systematic Reviews and Meta-Analyses Protocols
PROSPERO: International prospective register of systematic reviews
