# Supplementary material for: Clear aligner’s adverse effects: A systematic review protocol
Source: PLoS One. 2024 May 2;19(5):e0302049. doi: 10.1371/journal.pone.0302049 (PMC11065214; doi:10.1371/journal.pone.0302049)
Supplement: S2 Appendix — (DOCX) [file pone.0302049.s002.docx]

**APPENDIX 2**

| **DATABASE** | **QUERY SEARCH** | **RESULTS** |
| --- | --- | --- |
| PUBMED | #1 AND #2 | 1119 |
| 2 | "Impact"[Title/Abstract] OR "Effect"[Title/Abstract] OR "Effects"[Title/Abstract] OR "Adverse"[Title/Abstract] OR "Event"[Title/Abstract] OR "Events"[Title/Abstract] OR "Outcome"[Title/Abstract] OR "Outcomes"[Title/Abstract] OR "Wounds"[Title/Abstract] OR "Wound"[Title/Abstract] OR "Injury"[Title/Abstract] OR "Injuries"[Title/Abstract] OR "Lesion"[Title/Abstract] OR "Lesions"[Title/Abstract] OR "Trauma"[Title/Abstract] OR "Traumas"[Title/Abstract] OR "Fatigue"[Title/Abstract] OR "Burning"[Title/Abstract] OR "Ulcerations"[Title/Abstract] OR "Ulcer"[Title/Abstract] OR "Ulceration"[Title/Abstract] OR "Mouth"[Title/Abstract] OR "Oral"[Title/Abstract] OR "Tooth"[Title/Abstract] OR "Teeth"[Title/Abstract] OR "Tongue"[Title/Abstract] OR "Buccal"[Title/Abstract] OR "Periodontal"[Title/Abstract] OR "Gingival"[Title/Abstract] OR "Mucogingival"[Title/Abstract] OR "Gum"[Title/Abstract] OR "Gums"[Title/Abstract] OR "palate"[Title/Abstract] OR "soft tissues"[Title/Abstract] OR "Lip"[Title/Abstract] OR "Lips"[Title/Abstract] OR "Discomfort"[Title/Abstract] OR "Pressure"[Title/Abstract] OR "Pain"[Title/Abstract] OR "Black Spaces"[Title/Abstract] OR "Black Triangles"[Title/Abstract] OR "Embrasure"[Title/Abstract] OR "Caries"[Title/Abstract] OR "Biting"[Title/Abstract] OR "Chewing"[Title/Abstract] OR "Salivation"[Title/Abstract] OR "Bruxism"[Title/Abstract] OR "Dental Plaque"[Title/Abstract] OR "Biofilm"[Title/Abstract] OR "Microflora"[Title/Abstract] OR "Bacteria"[Title/Abstract] OR "Pathogen"[Title/Abstract] OR "Microorganism"[Title/Abstract] OR "Microbioma"[Title/Abstract] OR "Root Resorption"[Title/Abstract] OR "Root Resorptions"[Title/Abstract] OR "Speech"[Title/Abstract] OR "Misarticulation"[Title/Abstract] OR "Communication"[Title/Abstract] OR "Hygiene"[Title/Abstract] OR "Nausea"[Title/Abstract] OR "Swallowing"[Title/Abstract] OR "Breathing"[Title/Abstract] OR "Sore Throat"[Title/Abstract] OR "Swollen Throat"[Title/Abstract] OR "Anaphylaxis"[Title/Abstract] OR "Airway"[Title/Abstract] OR "Cough"[Title/Abstract] OR "Headache"[Title/Abstract] OR "Headaches"[Title/Abstract] OR "Sleep"[Title/Abstract] OR "Mastication"[Title/Abstract] OR "eat"[Title/Abstract] OR "Extraction"[Title/Abstract] OR "Extractions"[Title/Abstract] OR Endod*[Title/Abstract] OR "Root Canal "[Title/Abstract] OR "Pulpitis"[Title/Abstract] | 14136056 |
| 1 | "Invisalign"[Title/Abstract] OR "invisible appliance"[Title/Abstract] OR "invisible appliances"[Title/Abstract] OR "aligners"[Title/Abstract] OR "aligner"[Title/Abstract] | 2026 |
| EMBASE | ('Invisalign' OR 'invisible appliance' OR 'invisible appliances' OR 'aligners' OR 'aligner') AND ('Impact' OR 'Effect' OR 'Effects' OR 'Adverse' OR 'Event' OR 'Events' OR 'Outcome' OR 'Outcomes' OR 'Wounds' OR 'Wound' OR 'Injury' OR 'Injuries' OR 'Lesion' OR 'Lesions' OR 'Trauma' OR 'Traumas' OR 'Fatigue' OR 'Burning' OR 'Ulcerations' OR 'Ulcer' OR 'Ulceration' OR 'Mouth' OR 'Oral' OR 'Tooth' OR 'Teeth' OR 'Tongue' OR 'Buccal' OR 'Periodontal' OR 'Gingival' OR 'Mucogingival' OR 'Gum' OR 'Gums' OR 'palate' OR 'soft tissues' OR 'Lip' OR 'Lips' OR 'Discomfort' OR 'Pressure' OR 'Pain' OR 'Black Spaces' OR 'Black Triangles' OR 'Embrasure' OR 'Caries' OR 'Biting' OR 'Chewing' OR 'Salivation' OR 'Bruxism' OR 'Dental Plaque' OR 'Biofilm' OR 'Microflora' OR 'Bacteria' OR 'Pathogen' OR 'Microorganism' OR 'Microbioma' OR 'Root Resorption' OR 'Root Resorptions' OR 'Speech' OR 'Misarticulation' OR 'Communication' OR 'Hygiene' OR 'Nausea' OR 'Swallowing' OR 'Breathing' OR 'Sore Throat' OR 'Swollen Throat' OR 'Anaphylaxis' OR 'Airway' OR 'Cough' OR 'Headache' OR 'Headaches' OR 'Sleep' OR 'Mastication' OR 'eat' OR 'Extraction' OR 'Extractions' OR 'Endod*' OR 'Root Canal ' OR 'Pulpitis') | 1613 |
| LILACS | ('Invisalign' OR 'invisible appliance' OR 'invisible appliances' OR 'aligners' OR 'aligner' OR 'aparelho invisível' OR 'aparelhos invisíveis' OR 'alinhadores' OR 'alinhador') AND ('Impact' OR 'Impacto' OR 'Effect' OR 'Effects' OR 'Efeito' OR 'Efeitos' OR 'Adverse' OR 'Adverso' OR 'Adversos' OR 'Event' OR 'Events' OR 'Evento' OR 'Eventos' OR 'Outcome' OR 'Outcomes' OR 'Resultado' OR 'Resultados' OR 'Wounds' OR 'Wound' OR 'Injury' OR 'Injuries' OR 'Lesion' OR 'Lesions' OR 'Feridas' OR 'Ferida' OR 'Lesão' OR 'Lesões' OR 'Trauma' OR 'Traumas' OR 'Fatigue' OR 'Fadiga' OR 'Burning' OR 'Queimação' OR 'Ulcerations' OR 'Ulcer' OR 'Ulceration' OR 'Ulcerações' OR 'Úlcera' OR 'Úlceração' OR 'Mouth' OR 'Boca' OR 'Oral' OR 'Tooth' OR 'Teeth' OR 'Dente' OR 'Dentes' OR 'Tongue' OR 'Língua' OR 'Buccal' OR 'Bucal' OR 'Periodontal' OR 'Gingival' OR 'Mucogingival' OR 'Gengival' OR 'Mucogengival' OR 'Gum' OR 'Gums' OR 'Gengiva' OR 'Gengivas' OR 'palate' OR 'palato' OR 'soft tissues' OR 'tecidos moles' OR 'Lip' OR 'Lips' OR 'Lábio' OR 'Lábios' OR 'Discomfort' OR 'Desconforto' OR 'Pressure' OR 'Pressão' OR 'Pain' OR 'Dor' OR 'Black Spaces' OR 'Black Triangles' OR 'Espaços Negros' OR 'Triângulos Negros' OR 'Embrasure' OR 'Caries' OR 'Cárie' OR 'Biting' OR 'Mordida' OR 'Chewing' OR 'Mastigação' OR 'Salivation' OR 'Salivação' OR 'Bruxism' OR 'Bruxismo' OR 'Dental Plaque' OR 'Placa Dentária' OR 'Biofilm' OR 'Biofilme' OR 'Microflora' OR 'Bacteria' OR 'Bactéria' OR 'Pathogen' OR 'Patógeno' OR 'Microorganism' OR 'Microorganismo' OR 'Microbioma' OR 'Root Resorption' OR 'Root Resorptions' OR 'Reabsorção Radicular' OR 'Reabsorções Radiculares' OR 'Speech' OR 'Fala' OR 'Misarticulation' OR 'Más Articulaciones' OR 'Communication' OR 'Comunicación' OR 'Hygiene' OR 'Higiene' OR 'Nausea' OR 'Náusea' OR 'Swallowing' OR 'Deglución' OR 'Breathing' OR 'Respiración' OR 'Sore Throat' OR 'Dolor de Garganta' OR 'Swollen Throat' OR 'Garganta Inflamada' OR 'Anaphylaxis' OR 'Anafilaxia' OR 'Airway' OR 'Vía Aérea' OR 'Cough' OR 'Tosse' OR 'Headache' OR 'Headaches' OR 'Dolor de Cabeza' OR 'Dolores de Cabeza' OR 'Sleep' OR 'Sueño' OR 'Mastication' OR 'Masticación' OR 'eat' OR 'Comer' OR 'Extraction' OR 'Extractions' OR 'Extracción' OR 'Extracciones' OR 'Endod*' OR 'Root Canal ' OR 'Pulpitis' OR 'Endod*' OR 'Tratamiento de Conducto' OR 'Pulpitis') | 1349 |
| LIVIVO | ("Invisalign"[Title/Abstract] OR "invisible appliance"[Title/Abstract] OR "invisible appliances"[Title/Abstract] OR "aligners"[Title/Abstract] OR "aligner"[Title/Abstract]) AND ("Impact"[Title/Abstract] OR "Effect"[Title/Abstract] OR "Effects"[Title/Abstract] OR "Adverse"[Title/Abstract] OR "Event"[Title/Abstract] OR "Events"[Title/Abstract] OR "Outcome"[Title/Abstract] OR "Outcomes"[Title/Abstract] OR "Wounds"[Title/Abstract] OR "Wound"[Title/Abstract] OR "Injury"[Title/Abstract] OR "Injuries"[Title/Abstract] OR "Lesion"[Title/Abstract] OR "Lesions"[Title/Abstract] OR "Trauma"[Title/Abstract] OR "Traumas"[Title/Abstract] OR "Fatigue"[Title/Abstract] OR "Burning"[Title/Abstract] OR "Ulcerations"[Title/Abstract] OR "Ulcer"[Title/Abstract] OR "Ulceration"[Title/Abstract] OR "Mouth"[Title/Abstract] OR "Oral"[Title/Abstract] OR "Tooth"[Title/Abstract] OR "Teeth"[Title/Abstract] OR "Tongue"[Title/Abstract] OR "Buccal"[Title/Abstract] OR "Periodontal"[Title/Abstract] OR "Gingival"[Title/Abstract] OR "Mucogingival"[Title/Abstract] OR "Gum"[Title/Abstract] OR "Gums"[Title/Abstract] OR "palate"[Title/Abstract] OR "soft tissues"[Title/Abstract] OR "Lip"[Title/Abstract] OR "Lips"[Title/Abstract] OR "Discomfort"[Title/Abstract] OR "Pressure"[Title/Abstract] OR "Pain"[Title/Abstract] OR "Black Spaces"[Title/Abstract] OR "Black Triangles"[Title/Abstract] OR "Embrasure"[Title/Abstract] OR "Caries"[Title/Abstract] OR "Biting"[Title/Abstract] OR "Chewing"[Title/Abstract] OR "Salivation"[Title/Abstract] OR "Bruxism"[Title/Abstract] OR "Dental Plaque"[Title/Abstract] OR "Biofilm"[Title/Abstract] OR "Microflora"[Title/Abstract] OR "Bacteria"[Title/Abstract] OR "Pathogen"[Title/Abstract] OR "Microorganism"[Title/Abstract] OR "Microbioma"[Title/Abstract] OR "Root Resorption"[Title/Abstract] OR "Root Resorptions"[Title/Abstract] OR "Speech"[Title/Abstract] OR "Misarticulation"[Title/Abstract] OR "Communication"[Title/Abstract] OR "Hygiene"[Title/Abstract] OR "Nausea"[Title/Abstract] OR "Swallowing"[Title/Abstract] OR "Breathing"[Title/Abstract] OR "Sore Throat"[Title/Abstract] OR "Swollen Throat"[Title/Abstract] OR "Anaphylaxis"[Title/Abstract] OR "Airway"[Title/Abstract] OR "Cough"[Title/Abstract] OR "Headache"[Title/Abstract] OR "Headaches"[Title/Abstract] OR "Sleep"[Title/Abstract] OR "Mastication"[Title/Abstract] OR "eat"[Title/Abstract] OR "Extraction"[Title/Abstract] OR "Extractions"[Title/Abstract] OR Endod*[Title/Abstract] OR "Root Canal "[Title/Abstract] OR "Pulpitis"[Title/Abstract]) | 44 |
| SCOPUS | TITLE-ABS-KEY ("Invisalign" OR "invisible appliance" OR "invisible appliances" OR "aligners" OR "aligner") AND TITLE-ABS-KEY ("Impact" OR "Effect" OR "Effects" OR "Adverse" OR "Event" OR "Events" OR " Outcome" OR "Outcomes" OR "Wounds" OR "Wound" OR "Injury" OR "Injuries" OR "Lesion" OR "Lesions" OR "Trauma" OR "Traumas" OR "Fatigue" OR "Burning" OR "Ulcerations" OR "Ulcer" OR "Ulceration" OR "Mouth" OR "Oral" OR "Tooth" OR "Teeth" OR "Tongue" OR "Buccal" OR "Periodontal" OR "Gingival" OR "Mucogingival" OR "Gum" OR "Gums" OR "palate" OR "soft tissues" OR "Lip" OR "Lips" OR "Discomfort" OR "Pressure" OR "Pain" OR "Black Spaces" OR "Black Triangles" OR "Embrasure" OR "Caries" OR "Biting" OR "Chewing" OR "Salivation" OR "Bruxism" OR "Dental Plaque" OR "Biofilm" OR "Microflora" OR "Bacteria" OR "Pathogen" OR "Microorganism" OR "Microbioma" OR "Root Resorption" OR "Root Resorptions" OR "Speech" OR "Misarticulation" OR "Communication" OR "Hygiene" OR "Nausea" OR "Swallowing" OR "Breathing" OR "Sore Throat" OR "Swollen Throat" OR "Anaphylaxis" OR "Airway" OR "Cough" OR "Headache" OR "Headaches" OR "Sleep" OR "Mastication" OR "eat" OR "Extraction" OR "Extractions" OR endod* OR "Root Canal " OR "Pulpitis") | 1969 |
| Web of Science | TS=("Invisalign" OR "invisible appliance" OR "invisible appliances" OR "aligners" OR "aligner") AND TS=("Impact" OR "Effect" OR "Effects" OR "Adverse" OR "Event" OR "Events" OR "Outcome" OR "Outcomes" OR "Wounds" OR "Wound" OR "Injury" OR "Injuries" OR "Lesion" OR "Lesions" OR "Trauma" OR "Traumas" OR "Fatigue" OR "Burning" OR "Ulcerations" OR "Ulcer" OR "Ulceration" OR "Mouth" OR "Oral" OR "Tooth" OR "Teeth" OR "Tongue" OR "Buccal" OR "Periodontal" OR "Gingival" OR "Mucogingival" OR "Gum" OR "Gums" OR "palate" OR "soft tissues" OR "Lip" OR "Lips" OR "Discomfort" OR "Pressure" OR "Pain" OR "Black Spaces" OR "Black Triangles" OR "Embrasure" OR "Caries" OR "Biting" OR "Chewing" OR "Salivation" OR "Bruxism" OR "Dental Plaque" OR "Biofilm" OR "Microflora" OR "Bacteria" OR "Pathogen" OR "Microorganism" OR "Microbioma" OR "Root Resorption" OR "Root Resorptions" OR "Speech" OR "Misarticulation" OR "Communication" OR "Hygiene" OR "Nausea" OR "Swallowing" OR "Breathing" OR "Sore Throat" OR "Swollen Throat" OR "Anaphylaxis" OR "Airway" OR "Cough" OR "Headache" OR "Headaches" OR "Sleep" OR "Mastication" OR "eat" OR "Extraction" OR "Extractions" OR "Endod*" OR "Root Canal" OR "Pulpitis") | 1394 |
| Proquest | summary("Invisalign" OR "invisible appliance" OR "invisible appliances" OR "aligners" OR "aligner") AND summary("Impact" OR "Effect" OR "Effects" OR "Adverse" OR "Event" OR "Events" OR "Outcome" OR "Outcomes" OR "Wounds" OR "Wound" OR "Injury" OR "Injuries" OR "Lesion" OR "Lesions" OR "Trauma" OR "Traumas" OR "Fatigue" OR "Burning" OR "Ulcerations" OR "Ulcer" OR "Ulceration" OR "Mouth" OR "Oral" OR "Tooth" OR "Teeth" OR "Tongue" OR "Buccal" OR "Periodontal" OR "Gingival" OR "Mucogingival" OR "Gum" OR "Gums" OR "palate" OR "soft tissues" OR "Lip" OR "Lips" OR "Discomfort" OR "Pressure" OR "Pain" OR "Black Spaces" OR "Black Triangles" OR "Embrasure" OR "Caries" OR "Biting" OR "Chewing" OR "Salivation" OR "Bruxism" OR "Dental Plaque" OR "Biofilm" OR "Microflora" OR "Bacteria" OR "Pathogen" OR "Microorganism" OR "Microbioma" OR "Root Resorption" OR "Root Resorptions" OR "Speech" OR "Misarticulation" OR "Communication" OR "Hygiene" OR "Nausea" OR "Swallowing" OR "Breathing" OR "Sore Throat" OR "Swollen Throat" OR "Anaphylaxis" OR "Airway" OR "Cough" OR "Headache" OR "Headaches" OR "Sleep" OR "Mastication" OR "eat" OR "Extraction" OR "Extractions" OR "Endod*" OR "Root Canal" OR "Pulpitis") | 612 |
| Google Scholar | ("Invisalign" OR "invisible appliance" OR "invisible appliances" OR "aligners" OR "aligner") AND ("Impact" OR "Effect" OR "Effects" OR "Adverse" OR "Event" OR "Events" OR "Outcome" OR "Outcomes" OR "Wounds" OR "Wound" OR "Injury" OR "Injuries" OR "Lesion" OR "Lesions" OR "Trauma" OR "Traumas" OR "Fatigue" OR "Burning" OR "Ulcerations" OR "Ulcer" OR "Ulceration" OR "Mouth" OR "Oral" OR "Tooth" OR "Teeth" OR "Tongue" OR "Buccal" OR "Periodontal" OR "Gingival" OR "Mucogingival" OR "Gum" OR "Gums" OR "palate" OR "soft tissues" OR "Lip" OR "Lips" OR "Discomfort" OR "Pressure" OR "Pain" OR "Black Spaces" OR "Black Triangles" OR "Embrasure" OR "Caries" OR "Biting" OR "Chewing" OR "Salivation" OR "Bruxism" OR "Dental Plaque" OR "Biofilm" OR "Microflora" OR "Bacteria" OR "Pathogen" OR "Microorganism" OR "Microbioma" OR "Root Resorption" OR "Root Resorptions" OR "Speech" OR "Misarticulation" OR "Communication" OR "Hygiene" OR "Nausea" OR "Swallowing" OR "Breathing" OR "Sore Throat" OR "Swollen Throat" OR "Anaphylaxis" OR "Airway" OR "Cough" OR "Headache" OR "Headaches" OR "Sleep" OR "Mastication" OR "eat" OR "Extraction" OR "Extractions" OR "Endod*" OR "Root Canal" OR "Pulpitis") | 100 |
